# Peripartum Cardiomyopathy and HELLP Syndrome in a Primigravida: A Rare Case of Dual Challenges

**DOI:** 10.34763/jmotherandchild.20252901.d-25-00009

**Published:** 2025-08-16

**Authors:** Aditya Kalwaghe, Vijaykumar Barge, Vaishali Bramhanalkar, Rishikesh Kololikar, Shubhashish Singh

**Affiliations:** Department of Internal Medicine, Government Medical College & Hospital, Miraj, Maharashtra, India

**Keywords:** Peripartum cardiomyopathy, HELLP syndrome, Preeclampsia, Primigravida, Stillbirth

## Abstract

Peripartum cardiomyopathy (PPCM) and HELLP syndrome are rare, life-threatening pregnancy-associated conditions. PPCM, a dilated cardiomyopathy, presents with heart failure during the peripartum period. In comparison, HELLP syndrome involves hemolysis, elevated liver enzymes, and low platelet count. Symptoms of these conditions often mimic normal physiological discomforts and can be confused with typical peripartum issues. We report a unique case of a 24-year-old primigravida presenting with PPCM and HELLP syndrome. Timely diagnosis and multidisciplinary management resulted in her recovery, owing to a high level of clinical suspicion for her symptoms. Six-month follow-up showed normalisation of her cardiac function and complete clinical resolution.

## Introduction

Peripartum cardiomyopathy (PPCM) is a rare, life-threatening form of dilated cardiomyopathy causing heart failure in the absence of any identifiable cause of heart failure before the last month of pregnancy [1]. PPCM commonly occurs in women during their final month of pregnancy or within five months postpartum, with a peak incidence around the 35th week of pregnancy [1,2]. It is associated with several risk factors, such as preeclampsia, multiple pregnancies, advanced maternal age, and prolonged tocolytic use [2]. PPCM typically manifests with symptoms of congestive heart failure, such as dyspnea, orthopnea, and lower limb oedema [3].

HELLP syndrome, a severe complication of hypertensive disorders in pregnancy, is characterised by hemolysis, elevated liver enzymes, and low platelet count [4]. It increases the risk of fetal complications such as preterm birth, intrauterine growth restriction (IUGR), and even stillbirth, as well as maternal complications such as disseminated intravascular coagulation (DIC), acute kidney injury (AKI), placental abruption, pulmonary oedema, and even maternal mortality [5]. The syndrome commonly presents with right upper quadrant, epigastric pain, nausea, vomiting, and headaches [6]. The pathogenesis is linked to abnormal placentation and faulty immune tolerance, causing damage to fetal trophoblasts during the first trimester, along with an excessive inflammatory response [7].

Here, we discuss our experience in diagnosing and managing a patient presenting with both peripartum cardiomyopathy and HELLP syndrome, an extremely rare co-presentation. To the best of our knowledge, only seven such cases have been reported in medical literature.

## Case report

A 24-year-old primigravida at 31 weeks of gestation with uncontrolled gestational hypertension presented with epigastric pain, headache, and nausea. She has no significant past medical or family history. She reported absent fetal movement for two days, and handheld Doppler confirmed no fetal heart sounds. Ultrasonography (USG) confirmed intrauterine fetal demise with fetal parameters corresponding to 29 weeks of gestation and severe oligohydramnios (amniotic fluid index (AFI) 4 cm). Her previous ultrasound at 28 weeks showed a live fetus, adequate amniotic fluid (AFI = 12.8 cm), and an estimated fetal weight of 943 grams. Obstetric Doppler was normal, and the 19-week anomaly scan showed no fetal anomalies.

On examination, she had bilateral pedal oedema, mild icterus, and blood pressure of 166/102 mmHg. Lab results from admission bloodwork revealed mild anaemia, elevated liver enzymes, thrombocytopenia, elevated LDH, hyperbilirubinemia, elevated PT & INR, proteinuria, and schistocytes on peripheral smear, consistent with HELLP syndrome per the Tennessee classification (**Table 1**). She was induced with mifepristone, misoprostol, and Foleys catheter, followed by oxytocin infusion. IV labetalol was given to manage her hypertension and later transitioned to oral labetalol 200 mg eight-hourly. During labour, she received four units of random donor platelets (RDP) and four units of fresh frozen plasma (FFP), and IV furosemide. She delivered a stillborn fetus weighing 1.2 kg within 24 hours of induction, with signs of maceration.

**Table 1.** Laboratory values from admission day 1 to day 6.

**Laboratory parameters**	**Reference range**	**Admission – Day 1**	**Day 2**	**Day 3**	**Day 4**	**Day 5**	**Day 6**
Hemoglobin (gm/dL)	12 – 16	9.1	10.7	11.5	11.2	11.8	11.2
Total leucocyte count (/μL)	4000 – 10,000	14,000	15,200	12,700	10,200	9,500	9,700
Platelets (/μL)	150,000 – 450,000	28,000	48,000	81,000	106,000	160,000	169,000
Total bilirubin (mg/dL)	0 – 1.2	4.71	3.1	0.6	0.5	0.8	0.8
Direct bilirubin (mg/dL)	0 – 0.3	1.09	1.9	0.3	0.2	0.3	0.3
Indirect bilirubin (mg/dL)	0.2 – 0.8	3.62	1.2	0.3	0.3	0.5	0.5
LDH (IU/L)	140 – 271	728	-	-	-	-	-
Haptoglobin (mg/dL)	44 – 215	16	-	-	-	-	-
AST (IU/L)	0 – 40	446	362	221	53	30	22
ALT (IU/L)	0 – 32	234	288	146	51	37	29
ALP (IU/L)	39 – 117	268	262	191	101	110	88
PT (seconds)	9.3 – 12.5	17	12.5	11.6	-	-	-
INR	0.85 – 1.15	1.6	1.3	1.2	-	-	-
D-dimer (ng/dL)	< 500 ng/mL	1693	996	550	-	-	-

On day two, she developed dyspnea, tachypnea, sinus tachycardia, bilateral fine crepitations on auscultation, blood pressure of 148/98 mmHg and SpO2 of 87%. Chest X-ray was consistent with pulmonary oedema. She was transferred to the ICU and treated with supplemental oxygen, IV furosemide, two units of RDP, and one unit of FFP. Fluids were withheld, and strict monitoring of fluid input and output was implemented. Follow-up labs showed improvement (**Table 1**). Arterial blood gas analysis showed a pCO2 of 32 mmHg, a pO2 of 70 mmHg, a lactate level of 0.2 mmol/L, and a pH of 7.43. By day three, her symptoms improved, and her blood pressure decreased to 124/82 mmHg.

On day four, echocardiography revealed global left ventricular (LV) hypokinesia, mild systolic LV dilatation, and reduced LV ejection fraction (EF) of 40%, leading to a diagnosis of peripartum cardiomyopathy based on the 2021 European Society of Cardiology (ESC) guidelines for the Diagnosis and Treatment of Acute and Chronic Heart Failure. Labetalol was switched to lisinopril 10 mg and metoprolol 25 mg once daily. The patient was discharged on day eleven with stable vital signs.

At the six-month follow-up, her echocardiogram showed an LV EF recovery of 55%, and her blood pressure had normalised. The patient was maintained on metoprolol 25 mg once daily.

## Discussion

The maternal body undergoes significant physiological stress during pregnancy, resulting in a multitude of complex and adaptive anatomical and physiological changes [8]. The extent of these changes varies among individuals, making it very challenging to identify and diagnose abnormal adaptation and its associated disorders, especially in their early stages.

HELLP syndrome is a severe complication of preeclampsia, marked by intravascular platelet aggregation and widespread microvascular endothelial damage [5]. Around two-thirds of cases arise during pregnancy, predominantly between 27 and 37 weeks of gestation, while the remaining 30% develop after delivery [5]. Sibai BM reported signs and symptoms of HELLP syndrome in 15% of patients between 17 and 37 weeks of gestation, while 31 % experienced it after delivery and up to 6 days postdelivery [4]. Sibai BM et al. reported 33.3% fetal mortality mainly due to abruptio placentae and DIC [6].

Peripartum cardiomyopathy poses a serious risk with a mortality rate of 25 to 50% [1]. Preeclampsia is a key risk factor, potentially contributing to cardiac dysfunction through anti-angiogenic pathways, pregnancy-related hemodynamic stress, oxidative stress, and genetic predisposition [2]. A study by Demakis et al. reported persistent left ventricular dysfunction at the sixth month of follow-up in nearly 50% patients, and an 85% mortality rate among those within the next 5 years [9]. A study done by Elkayam et al. reported a higher likelihood of cardiac function normalisation in patients with an initial LV EF ≥ 30% [3].

This case posed significant diagnostic challenges due to the overlapping symptoms, such as dyspnea and pedal oedema, which are common during the peripartum period and not specific to HELLP syndrome or PPCM. Patient's initial presentation, along with her abnormal labs, prompted the diagnosis of HELLP syndrome. Concurrently, her dyspnea and pedal oedema, initially suspected as a result of fluid overload after transfusions, warranted further investigations, leading to a diagnosis of PPCM.

Delivery remains the cornerstone treatment for both HELLP syndrome and PPCM, as it alleviates stress on the maternal heart and accelerates recovery [10]. Bayraktaroğlu et al. reported resolution of the disease within 24 – 48 hours post-placenta delivery [10]. To ensure the safety and well-being of both the baby and the mother, the timing of delivery is determined based on the severity of the conditions, the clinical stability of the mother, and the stage of gestation [5]. Our patient's rapid recovery and normalisation of labs, along with complete normalisation of LV function at six-month follow-up, were likely due to early diagnosis and treatment, as well as timely delivery, which facilitated a rapid recovery. However, there is limited data exploring any connection between HELLP syndrome and cardiac structural or functional changes.

In conclusion, this case highlights the importance of maintaining a high index of suspicion in patients with hypertension and heart failure symptoms, given their overlapping symptoms with normal peripartum discomfort. Both conditions require timely diagnosis and multidisciplinary management to prevent serious complications. By presenting this rare co-presentation, we hope to enhance awareness and inspire further research into peripartum complications.
